# Upper lip metastasis of sarcomatoid carcinoma of the lung – an unusual site of disease: a case report

**DOI:** 10.1186/s13256-016-1178-y

**Published:** 2017-01-19

**Authors:** Tri Le, Melissa Mayer, Joseph Sailors, David E. Gerber, John M. Truelson

**Affiliations:** 1 Department of Internal Medicine, University of Texas Southwestern Medical Center, 5323 Harry Hines Blvd., Dallas, TX 75390 USA; 2 Harold C. Simmons Cancer Center, University of Texas Southwestern Medical Center, 2201 Inwood Road, 3rd Floor, Suite 500, Dallas, TX 75390-9125 USA; 3 Department of Pathology, University of Texas Southwestern Medical Center, 5323 Harry Hines Blvd., Dallas, TX 75390 USA; 4 Department of Otolaryngology, University of Texas Southwestern Medical Center, 5323 Harry Hines Blvd., Dallas, TX 75390 USA

**Keywords:** Lip, Cutaneous, Metastasis, Lung cancer, Sarcomatoid

## Abstract

**Background:**

Lip metastases are rare clinical events that are frequently mistaken for other diagnoses. For sarcomatoid lung carcinoma, a rare histologic variant of non-small cell lung cancer, the incidence and pattern of cutaneous spread is poorly understood.

**Case presentation:**

We present a case of a 79-year-old African American man with a rapidly progressive upper lip cutaneous lesion that provided the first evidence of distant metastatic spread of sarcomatoid lung carcinoma.

**Conclusions:**

This is the first reported case of lip metastasis in sarcomatoid lung carcinoma. It highlights the importance of maintaining a high level of suspicion for metastatic disease in the presence of new cutaneous findings as they may be the first evidence of advanced disease.

## Background

Sarcomatoid lung carcinoma is an aggressive and rare histologic variant of non-small cell lung cancer (NSCLC; 0.3 to 1.3% of all lung malignancies) that is morphologically defined by components of sarcoma or sarcoma-like differentiation (for example, spindles and/or giant cells) [1]. Prompt recognition of metastatic progression is critical to management, as staging determines treatment and prognosis. The skin provides a noninvasive opportunity to identify metastatic disease, but the pattern and incidence of cutaneous spread in this disease remains unknown. Here, we present a case of rapidly progressive upper lip cutaneous metastasis from a case of sarcomatoid lung carcinoma. To the best of our knowledge, it is the first documented case of lip metastasis in this disease, establishing the lip as a potential site of spread.

## Case presentation

**Fig. 1:**
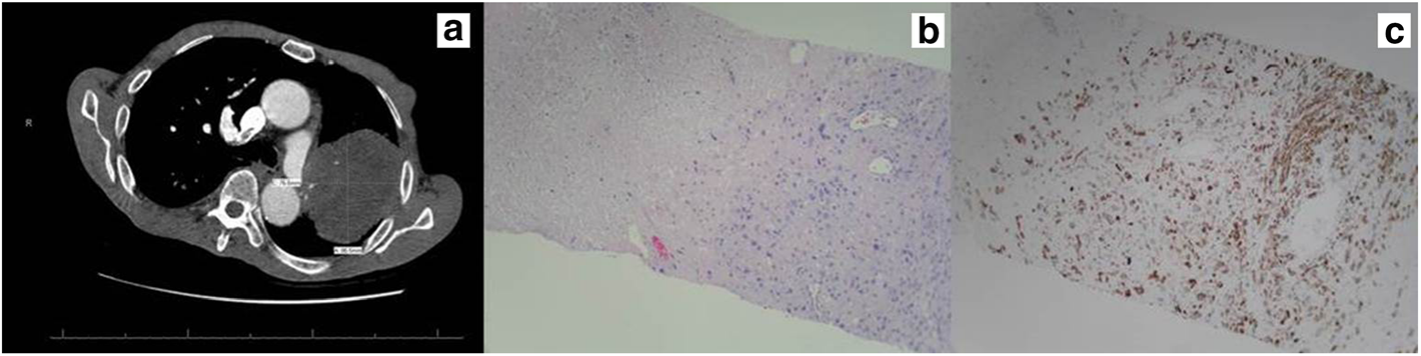
Primary sarcomatoid non-small cell lung cancer. **a** A 7.7×8.7 cm mass in the upper lobe of the left lung and the lingula causing extrinsic compression of the left pulmonary artery and its branches with extension into the left superior pulmonary vein, mediastinum, and probably the pericardium. **b** Core fragments of lung mass demonstrating spindle cell processes, large areas of necrosis, and intermediate-sized nuclei (100×). **c** Immunohistochemical staining with cells positive for CAM5.2 (100×)

A 79-year-old African American man with a history notable for heavy tobacco use, traumatic brain injury following a motor vehicle accident, hypothyroidism, and chronic lymphocytic leukemia last requiring therapy 4 years previously presented with 1 month of persistent cough. On a chest X-ray, a large mass in the mid-lung zone of his left lung was noted. Chest computed tomography (CT) demonstrated a mass in the upper lobe of his left lung measuring 7.7×8.7 cm and a 1.1 cm left hilar lymph node (Fig. 1a). A CT-guided percutaneous biopsy of the lung mass revealed malignant spindled and focally epithelioid neoplasm with extensive necrosis with poor differentiation favoring sarcomatoid carcinoma (Fig. 1b). Immunohistochemistry demonstrated positive staining for epithelial markers CAM5.2 (Fig. 1c), cytokeratin (CK) 5/6, CK903, and CK7, and negative staining for thyroid transcription factor 1 (TTF-1), p63, desmin, S100, and paired-box gene 8 (PAX8).

**Fig. 2:**
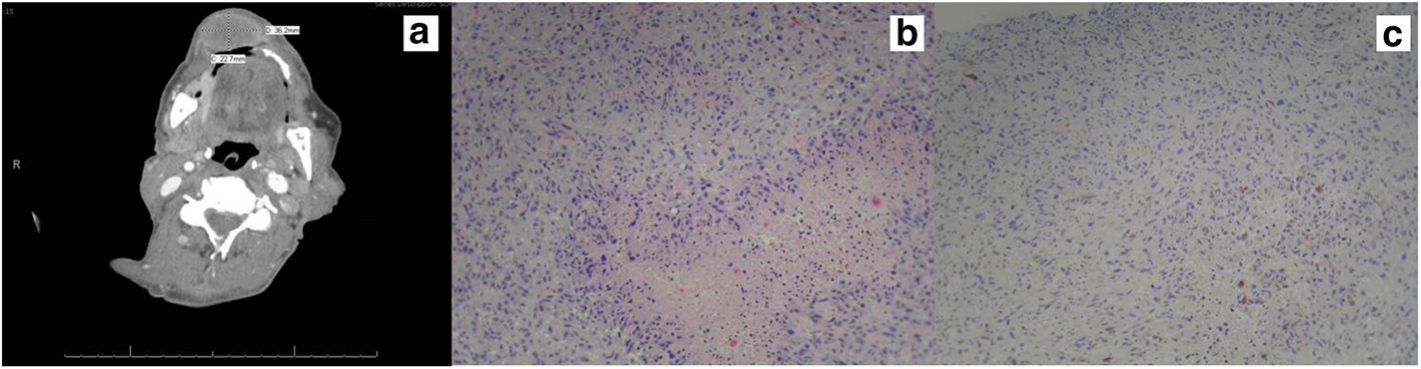
Sarcomatoid non-small cell lung cancer metastasis to upper lip. **a** A 2.1 cm anteroposterior × 3.6 cm transverse × 3.8 cm craniocaudal rim-enhancing mass involving the right upper lip with mild surrounding inflammatory changes. **b** Scant benign superficial epithelium with an overriding subepithelial spindle and epithelioid cell neoplastic population with intermediate-sized nuclei with some degeneration and moderate amounts of pink cytoplasm, scattered multinucleated giant cells, and small intratumoral foci of necrosis (100×). **c** Immunohistochemical staining with scattered MNF116-positive cells (100×)

At this time, he was also noted to have an ulcerated area on his upper lip on which topical anesthetic agents were placed. Seven days later, a follow-up examination revealed painful and substantial upper lip swelling. An abscess was suspected, systemic antibiotics were prescribed, and he was referred urgently to our Otolaryngology service. He was found to have a 2.7 cm upper lip mass with normal overlying skin. A CT of his neck revealed a 2.1 cm anteroposterior (AP) × 3.6 cm transverse × 3.8 cm craniocaudal (CC) right upper lip mass with surrounding inflammatory change (Fig. 2a). An incisional biopsy demonstrated metastatic sarcomatoid carcinoma, with morphologic and immunohistochemical characteristics similar to the primary lung lesion (Fig. 2b, c).

Subsequent imaging demonstrated two intracranial metastases. He received palliative radiation 3000 cGy in 10 fractions to his upper lip and 3000 cGy in 10 fractions to his brain. He continued to decline functionally; he was admitted to our in-patient hospice unit, and died 39 days after initial diagnosis of the primary sarcomatoid lung cancer.

## Discussion

Sarcomatoid lung carcinoma is an aggressive and rare histologic variant of NSCLC (0.3 to 1.3% of all lung malignancies) that is morphologically defined by components of sarcoma or sarcoma-like differentiation (for example, spindles and/or giant cells) [1]. There are five primary subgroups recognized under the 2004 and the 2015 *World Health Organization Classification of Tumors of the Lung*: pleomorphic carcinoma, spindle cell carcinoma, giant cell carcinoma, carcinosarcoma, and pulmonary blastoma [1–4]. Relative to other NSCLC histologies, sarcomatoid carcinoma has poorer stage-by-stage prognosis [3] and earlier recurrence after resection. The average age at diagnosis is 60 to 65 years [1, 3, 5–15]. Several studies suggested an increased male prevalence [1, 2, 4, 5, 8, 11], while others, including the largest epidemiologic study to date using the Surveillance, Epidemiology, and End Results database, suggested a nearly 1-to-1 gender ratio [3, 12]. Sarcomatoid carcinoma is associated with heavy tobacco smoking history [1, 9, 15]. Clinical symptoms are nonspecific (for example, cough, hemoptysis, dyspnea, thoracic pain, weight loss, fatigue, and fever due to recurrent pneumonia) and are often related to pulmonary tumor localization [1, 9, 11, 14].

In the limited experience reported for this aggressive disease, treatment varies significantly with stage. Local disease appears to have a good outcome with surgical resection alone [15]. In metastatic disease, systemic chemotherapy continues to be to be the mainstay of treatment. Unfortunately, the response to conventional NSCLC therapy is poorer than that of more common histologic variants [16]. Given the distinction in prognosis and treatment between local disease and distant spread, it is critical to identify metastatic disease early. Cutaneous involvement involving the face is a readily available, noninvasive opportunity for physicians to identify metastatic disease in the absence of sites of spread.

Malignancy involving the lip is a rare clinical event and largely represented by primary squamous cell (49%) and basal cell (40%) carcinomas. Other etiologies include salivary gland origin (9%) and metastatic cancer to the lip (2%) [17]. Among the few reported cases of lip metastases in the literature, lung, gastric adenocarcinoma, breast, lymphoma, and renal cell carcinoma are described [18]. Clinical manifestation is often a submucosal mass with intact overlying skin, occasionally with ulceration, as was observed in this case [19]. To the best of our knowledge, this is the first reported case of sarcomatoid carcinoma of the lung metastasizing to the lip. High suspicion for metastatic disease should be maintained when new skin findings are observed in patients with a known cancer diagnosis, with low threshold for early histologic confirmation by biopsy.

## Conclusions

This case establishes the lip as a potential site of metastatic spread for sarcomatoid lung carcinoma. Lip and other cutaneous metastases provide an easily identifiable opportunity to diagnose distant disease in patients with cancer diagnoses.

## Abbreviations

CK: Cytokeratin
CT: Computed tomography
NSCLC: Non-small cell lung cancer
